# TS-AMIR: a topology string alignment method for intensive rapid protein structure comparison

**DOI:** 10.1186/1748-7188-7-4

**Published:** 2012-02-15

**Authors:** Jafar Razmara, Safaai Deris, Sepideh Parvizpour

**Affiliations:** 1 Faculty of Computer Science and Information Systems, Universiti Teknologi Malaysia 81310, Johor Bahru, Malaysia; 2 Faculty of Bioscience and Bioengineering, Universiti Teknologi Malaysia 81310, Johor Bahru, Malaysia

## Abstract

**Background:**

In structural biology, similarity analysis of protein structure is a crucial step in studying the relationship between proteins. Despite the considerable number of techniques that have been explored within the past two decades, the development of new alternative methods is still an active research area due to the need for high performance tools.

**Results:**

In this paper, we present TS-AMIR, a Topology String Alignment Method for Intensive Rapid comparison of protein structures. The proposed method works in two stages: In the first stage, the method generates a topology string based on the geometric details of secondary structure elements, and then, utilizes an n-gram modelling technique over entropy concept to capture similarities in these strings. This initial correspondence map between secondary structure elements is submitted to the second stage in order to obtain the alignment at the residue level. Applying the Kabsch method, a heuristic step-by-step algorithm is adopted in the second stage to align the residues, resulting in an optimal rotation matrix and minimized RMSD. The performance of the method was assessed in different information retrieval tests and the results were compared with those of CE and TM-align, representing two geometrical tools, and YAKUSA, 3D-BLAST and SARST as three representatives of linear encoding schemes. It is shown that the method obtains a high running speed similar to that of the linear encoding schemes. In addition, the method runs about 800 and 7200 times faster than TM-align and CE respectively, while maintaining a competitive accuracy with TM-align and CE.

**Conclusions:**

The experimental results demonstrate that linear encoding techniques are capable of reaching the same high degree of accuracy as that achieved by geometrical methods, while generally running hundreds of times faster than conventional programs.

## Background

Today, biologists are faced with rapidly growing amounts of unknown sequence and structure data related to protein databases. Taking advantage of efficient analysis tools, biologists are highly motivated to derive biological insights from these biomolecules. Sequence comparison tools are commonly used to determine the similarities between proteins with a high degree of similarity, whereas structure comparison methods are essentially utilized to highlight the evolutionary relationships among proteins. Additionally, scientists consider the biological role for these macromolecules as being strongly dependent on their 3D-structure, which has attracted their interest to employ accurate and reliable structure comparison tools with respect to such molecules.

Considering the large amount of unknown data which exists in structural biology, efficient powerful tools are needed to investigate, analyze and classify the properties and functionalities of this data. Furthermore, several tools are available for structural analysis and comparison of biological data. The lack of a universal measurement standard for the evaluation of these methods has persuaded biologists to use different tools and scoring functions in their inquiries. This diversity has provided facilities for biologists to extract their required information for query data more efficiently.

In the structure comparison problem, determining the structural alignment of a protein pair is a fundamental step. The simplest case of the problem occurs when an initial correspondence map between the residue pairs is provided by a sequence alignment procedure. However, providing this initial correspondence map is not possible for protein pairs with low sequence identity. Thus, the methods generally involve comparisons without any prior specified correspondence between residues. Therefore, there is a problem finding the optimal alignment of two structures in unbounded dimensions known as NP-hard [1]. Many algorithms have been developed using heuristic strategies to compare geometrical coordinates of the C_α _backbone atoms in order to find the best optimal correspondence between residue pairs. The techniques include distance matrices comparison (DALI) [2], vector alignment of secondary structure alignment (VAST) [3], combinatorial extension (CE) [4], matching molecular models obtained from theory (MAMMOTH) [5], secondary structure matching (SSM) [6], dynamic programming on TM_score _rotation matrix (TM-align) [7], genetic algorithm for non-sequential gapped protein structure alignment (GANGSTA) [8] and many others ([9-12]). Several comprehensive reviews and evaluation of the methods have been reported in literatures ([13-15]).

In order to overcome the complexity of the structure comparison problem and the difficulties of searching a large structure database, existing methods commonly proceed to represent protein structure in a summarized form. Recently, various methods have been explored to model a 3D-structure of protein in a linear 1D-sequence, in which known sequence alignment tools like FASTA [16], BLAST [17] or other language modelling techniques are employed to capture structural similarities among proteins. The methods include protein structure modelling in a set of topology strings (TOPSCAN) [18], hidden Markov model derived structural alphabet (SA-Search) [19], representing discrete internal angles of protein backbone as a sequence (YAKUSA) [20], kappa-alpha (κ, α) plot derived structural alphabet and BLOSUM-like substitution matrix (3D-BLAST) [21], Structural similarity search by Ramachandran codes (SARST) [22], structure-to-string translator and a hash table to store n-grams (Lajolla) [23] and protein structure representation as a bag-of-words of backbone fragments (FragBag) [24]. Linear encoding techniques adopted from these methods commonly reduce running time of the algorithms as they run hundreds of times faster than geometrical methods like CE [21,22]. Additionally, sequence-based schemes are more relevant to be extended for use in multiple structure alignment, fold recognition and genomic annotation studies [22]. However, 3D-structure conversion into 1D-sequence leads to lose some of the structural details of proteins. Consequently, these methods obtain lower alignment accuracy when compared to highly accurate geometrical search tools. Accordingly, it is a challenging task to develop an algorithm with which the speed advantage of linear encoding techniques with regard to their competitive alignment accuracy can be gained.

Recently, a novel text modelling approach has been developed by this group [25] in which the structural comparison and alignment of biomolecules can be carried out. The algorithm summarizes protein 3D-structure in a textual sequence and applies a cross-entropy concept over n-gram modelling in order to capture similarities between protein sequences. Considering the fruitful results of this method in terms of accuracy and running speed, an extension of the approach, hereby named TS-AMIR, is introduced to be used for secondary structure modelling in a topology string with the ultimate goal of developing a structural protein alignment tool. TS-AMIR is a simple and fast method with comparable accuracy to state of the art programs.

## Methods

The TS-AMIR algorithm works in two stages. In the first stage, a correspondence map is made between secondary structure elements of two compared structures using text modelling techniques. The procedure makes a topology string based on the geometry of the secondary structure elements of each structure followed by the application of the n-gram modelling technique to find the best matching condition between two structures. The second stage uses a heuristic step-by-step algorithm to make an alignment at the residue level by calculating a rotation matrix derived from applying the method suggested by Kabsch [26,27]. Detailed explanations of the method are described in the following sections.

### Secondary structure modelling in a topology string

**Figure 1:**
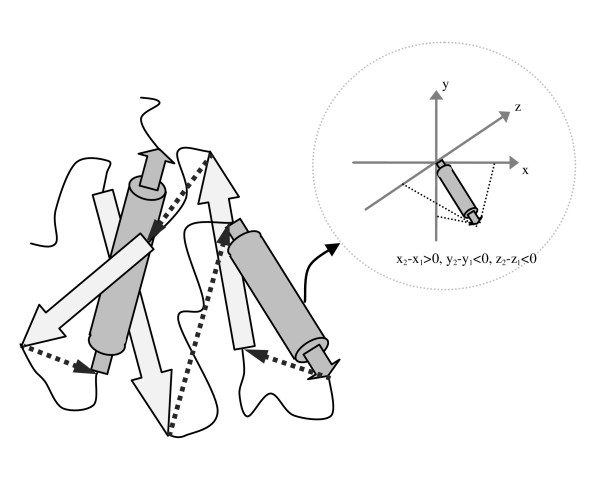
**Secondary structure elements representation as vectors in 3D-space**. Dashed vectors represent inter-SSEs vectors.

**Figure 2:**
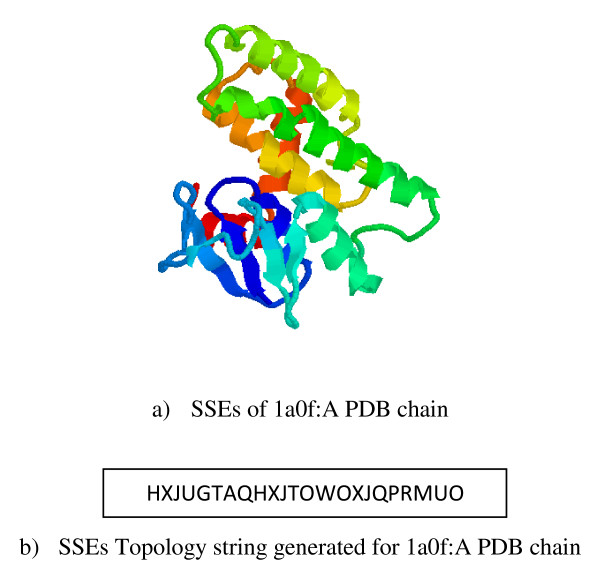
**A typical example for secondary structure modelling in a topology string**.

In order to reduce the complexity of the structure comparison problem, the methods most commonly use a simplified representation of the protein backbone structure. The secondary structure constitutes the backbone of a protein which is essentially made of highly regular substructures of α-helices and β-strands. Having secondary structure elements (SSEs) of a protein extracted from its PDB file, TS-AMIR summarizes SSEs in a topology string based on the direction of each SSE vector in 3D-space. To do this, each SSE is represented as a vector by ***r***_*SSE *_= ***r***_*b *_- ***r***_*e *_[28] where:

(1) $$\begin{array}{c} r_{b}=\left(0.74r_{i}+r_{i+1}+r_{i+2}+0.74r_{i+3}\right)/3.48,\\ r_{e}=\left(0.74r_{j-3}+r_{j-2}+r_{j-1}+0.74r_{j}\right)/3.48 \end{array}$$

for helices and

(2) $$\begin{array}{c} r_{b}=\left(r_{i}+r_{i+1}\right)/2,\\ r_{e}=\left(r_{j-1}+r_{j}\right)/2 \end{array}$$

for strands (indices *i *and *j *denote the first and last residues in each element). Then, each SSE vector is encoded to a letter within a string based on the sign of its *x, y *and *z *components as shown in Table 1. In addition to the SSE vectors, the method also assumes intermediate vectors between the end and start points of two consecutive SSE vectors respectively. These inter-SSEs vectors, represented as dashed vectors in Figure 1, reflect the relative position of each SSE vector with respect to its previous and next SSE vectors in 3D-coordinates in the topology string. Accordingly, the spatial locations of secondary structure elements are modelled in the topology string in which each letter denotes an SSE vector or inter-SSEs vector. Figure 2 shows a typical example for secondary structure modelling in the topology string.

**Table 1:** Defined labels for secondary structure vectors

	Vector type		
Direction*	**Strand**	**Helix**	**Inter-SSEs**

*x_2_-x_1 _*> 0, *y_2_-y_1 _*> 0, *z_2_-z_1 _*> 0	A	I	Q
*x_2_-x_1 _*> 0, *y_2_-y_1 _*> 0, *z_2_-z_1 _*< 0	B	J	R
*x_2_-x_1 _*> 0, *y_2_-y_1 _*< 0, *z_2_-z_1 _*> 0	C	K	S
*x_2_-x_1 _*> 0, *y_2_-y_1 _*< 0, *z_2_-z_1 _*< 0	D	L	T
*x_2_-x_1 _*< 0, *y_2_-y_1 _*> 0, *z_2_-z_1 _*> 0	E	M	U
*x_2_-x_1 _*< 0, *y_2_-y_1 _*> 0, *z_2_-z_1 _*< 0	F	N	V
*x_2_-x_1 _*< 0, *y_2_-y_1 _*< 0, *z_2_-z_1 _*> 0	G	O	W
*x_2_-x_1 _*< 0, *y_2_-y_1 _*< 0, *z_2_-z_1 _*< 0	H	P	X

* (x_1_, y_1_, z_1_) and (x_2_, y_2_, z_2_) denote start and end points of SSE vectors.

### Text similarity measurement by n-gram modelling

Having secondary structure modelling done in a topology string, a language modelling technique can be used to compare the strings in order to achieve an initial match between two protein structures. Several kinds of language modelling techniques have already been used to capture similarities among biological sequences. Markov chains are considered as the most fundamental approach used in language modelling and protein sequences similarity measurement [25,29]. In this model, the existence of a word *w_k _*at a location *k *in an input text depends upon its *n *previous words *w_k-n_,..., w_k-1_*. Due to the simplicity of the concept, the so called n-gram modelling has been used most often in formal linguistics studies [29]. Moreover, entropy is a useful concept accounting for quantifying the information in a textual sequence and making a connection with probabilistic language modelling. As described by Bogan-Marta et al. [29], entropy estimation indicates how a specific protein sequence is well predicted by a given model. To compare two sequences, cross-entropy measure is the relevant tool, where n-gram model is firstly made by counting the words of one sequence in the training phase, and then, the predictability of the second sequence is measured in the recall phase via formula:

(3) $$\begin{array}{ll} H\left(X,P_{M}\right) & =-\sum_{w_{i}^{n}}P\left(w_{i}^{n}\right)\text{log}\left(2+P_{M}\left(w_{i+n}|w_{i}^{n-1}\right)\right)\;\\ & =-\frac{1}{N}\sum_{w_{i}^{n}}Count\left(w_{i}^{n}\right)\text{log}\left(2+P_{M}\left(w_{i+n}|w_{i}^{n-1}\right)\right)\;\\ & \; \end{array}$$

where the variable *X *is in the form of n-gram represented by $w_{i}^{n}=\left\{w_{i},w_{i+1},\;...,\;w_{i+n-1}\right\}$ ranging over all the words of the first sequence. The summation runs over all the possible n-gram words $w_{i}^{n}$, and *N *is the number of n-grams. The term *P *($w_{i}^{n}$) results from the word count within the first sequence via $Count\left(w_{i}^{n}\right)$. Moreover, the conditional probability in the summation relates the *n*-th element of an n-gram with the preceding *n*-1 elements, which can be computed by counting the words of the second sequence and having the model estimated:

(4) $$P\left(w_{i+n}|w_{i}^{n-1}\right)=Count\left(w_{i+n}\right)/Count\left(w_{i}^{n-1}\right)$$

Moreover, the term 2 is added to the logarithm function in formula 3 in order to prevent loss of data when all the words are counted once and the conditional probability becomes zero.

The sequence comparison method models both sequences in the n-gram form, and also utilizes cross-entropy tool to measure their similarity via the formula:

(5) $$D\left(s_{q},S_{i}\right)=|H\left(X_{q},P_{M_{i}}\right)-PS|$$

where *PS *is the perfect score using the first sequence as reference and model sequences. *S_q _*and *S_i _*also denote the first and second sequences respectively. As a result, the lower value of *D *(*S_q_, S_i_*) indicates the higher similarity of the compared sequences. In order to cope with big differences in the length of two compared sequences, the technique considers the protein with lower length as the query protein.

The above introduced cross-entropy measure is used to compare the topology strings of two proteins resulting in finding the initial overlap between two structures according to a task described below.

### Secondary structure matching using topology string

**Figure 3:**
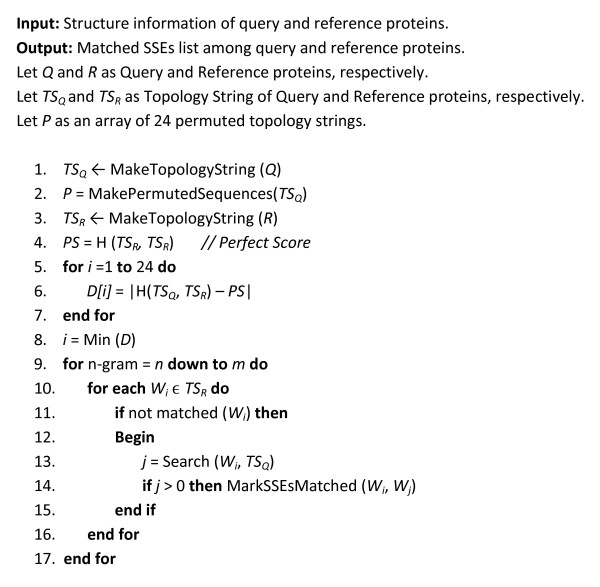
**The algorithm for secondary structure matching using topology strings**.

**Figure 4:**
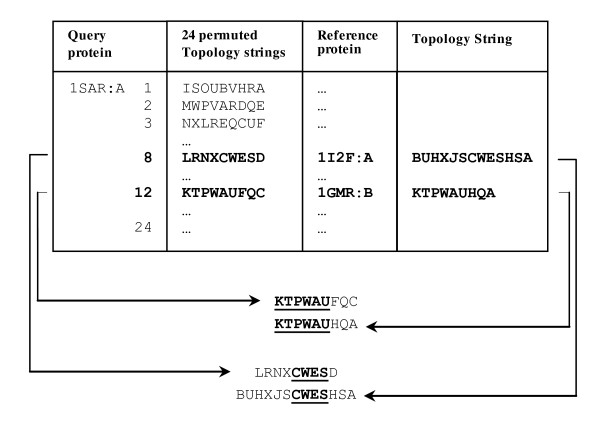
**An example for matching topology string of two reference proteins with 24 permuted topology strings of query protein**.

The 3D-coordinates of any pair of protein structures are available in an arbitrary relative orientation, in which the matched parts may not correspond in two structures. Accordingly, the structure comparison methods need coordinates with independent representation of the structures making them comparable. In order to obtain an initial match between two structures, our method applies an algorithm with respect to the scheme introduced by Martin [18]. Figure 3 is the algorithm which has been developed for matching SSEs using topology strings. To this end, the topology string of the query protein is permuted by rotating its structure 90 degrees around the *x, y *and *z *axes (line 2 in Figure 3). For each rotation around an axis, letters of the topology string are replaced according to Table 2. Therefore, 24 different secondary topology strings are created. Then, the above introduced cross-entropy measure is utilized to compare these 24 permuted topology strings of the query protein with the topology string of the reference protein, with which the most similar strings are chosen (lines 4-8 in Figure 3). In sequel, identical n-gram words of the two topology strings are marked as matched in an iterative procedure accounting for the decreasing size of n-grams starting from *m *(chosen empirically 6) down to the basic size of an n-gram (chosen at 3) (lines 9-17 in Figure 3). Figure 4 represents the SSE matching procedure for two sample secondary topology strings.

**Table 2:** Permutations on SSEs direction labels based on 90 degree rotation around axes

	Strand	Helix	Inter-SSEs
Old	A B C D E F G H	I J K L M N O P	Q R S T U V W X
Rotate 90° around *x *	B D A C F H E G	J L I K N P M O	R T Q S V X U W
Rotate 90° around *y *	E A G C F B H D	M I O K N J P L	U Q W S V R X T
Rotate 90° around *z *	E F A B G H C D	M N I J O P K L	U V Q R W X S T

In addition to the above procedure to match the identical words of two topology strings, the method makes another effort to match SSEs with semi-adjacent vectors, where they are different in the sign of only one of the *x, y *and *z *components. This can be done by making a group of semi-adjacent letters for each defined letter in Table 1. Table 3 represents this grouping scheme for the letters of strand type of SSEs. In this table, for example, the letter F is marked as semi-adjacent for the letters B, E and H. Similar groups of letters can be made for the helix and inter-SSE vectors.

**Table 3:** Semi-adjacent letters defined for Strand SSE vectors

	A	B	C	D	E	F	G
**H**				*		*	*

**G**			*		*		
	
**F**		*			*		
		
**E**	*						
			
**D**		*	*				
				
**C**	*						
					
**B**	*						

### Structure alignment at the residue level

The procedure introduced above for matching the SSEs provides the alignment map at the residue level. The map is submitted to a heuristic algorithm which utilizes the rotation matrix of Kabsch method [26,27] in an iterative procedure over selected pairs of residues. The following steps are done until the convergence of the alignment is fulfilled:

1) For each pair of the matched SSEs, put the start and end residues as temporarily aligned pairs in the alignment list. Then, compute and apply the rotation matrix to achieve an initial overlap between two structures.

2) For each pair of the matched SSEs, find *n *neighboring residues (*n *is chosen 3 for strands and 4 for helices [6]) having minimum distance, put them in the alignment list, and then, extend the alignment to the ends of the elements.

3) For unaligned residues between the aligned residues in the previous step, find contact pairs as defined by Krissinel and Henrick [6]. In this definition, two atoms *A *and *B *of chains 1 and 2 are considered as a contact pair if their distance is less than the distance between *A *and any other residues of chain 2 and also the distance between *B *and any other residues of chain 1. Start with the shortest contacts, extend the alignment to the rest of the residue pairs having a distance less than $d_{0}=1.24\sqrt[{3}]{L_{N}-15}-1.8$[30]. (d_0 _is a distance parameter to normalize distances and make the score independent of the protein size where L_N _is the length of the shorter protein).

4) Make and apply the rotation matrix based on the aligned pairs of residues in the alignment list.

5) Repeat steps 2, 3 and 4 until the rotation matrix converges.

In order to look for the next mapping residues in step 3, the method applies a recursive task considering a linear gap penalty. Moreover, in this procedure, a pair of residues could not be aligned if they belong to different secondary structure types.

Obviously, the alignment quality depends on the contradictory requirements of obtaining a higher length of alignment and a lower RMSD. TM_score _[30] is a reasonable single measure to assess the alignment quality by making a balance between the alignment length and accuracy according to the following formula:

(6) $$TM_{score}=Max\left[\frac{1}{L_{q}}\sum_{i=1}^{L_{n}}1/1+{\left(\frac{d_{i}}{1.24\sqrt[{3}]{L_{q}-15}-1.8}\right)}^{2}\right]$$

where L_n _and L_q _are the length of the alignment and query protein respectively, and d_i _is the distance between *i-*th pair of aligned residues. As described by Zhang and Skolnick [7], TM_score _always has a value between [0,1] where the higher value is better. Accordingly, we used TM_score _in order to investigate the alignment optimality, with which the optimal gap penalty is chosen. To this end, we used a randomly selected dataset of 1000 protein structures to test the alignment quality in different gap penalties based on evaluation of the TM_score_.

## Results

The above introduced algorithm, called TS-AMIR, was implemented in Microsoft Visual C++ using MS-Windows XP. This section reports the results of experiments in order to assess the performance of the method. The method was subjected to different datasets and its outputs were compared with CE [4] and TM-align [7] representing two powerful geometrical methods and YAKUSA [20], 3D-BLAST [21] and SARST [22] as three well-known linear encoding methods.

### Determination of optimal n-gram size

In order to determine the optimal size of n-gram in the SSEs sequence matching procedure and to balance its accuracy and sensitivity against computational efficiency, an accuracy index of similarity database search was adopted from Receiver Operating Characteristic (ROC) curves [31]. The index indicates true positive versus false positive rates in the ROC- curve for different initial sizes of the n-gram model. The goal is to obtain values where the true positive rates reach 1 or a very close value to 1, for a very small rate of the false positives. The experiment used a set of 90 proteins carefully selected from the SCOP database belonging to All Alpha, All Beta, Alpha and Beta and Alpha+Beta categories with less than 40% sequence identity, having more than 7 SSEs [32]. Moreover, the SCOP classification database was considered to be the gold standard.

Table 4 shows the values of true positive versus false positive rates for different initial sizes of the n-gram adopted in the SSEs sequence matching procedure. As shown in this table, the method reaches to the optimal true positive rate in the case of 6-gram while its false positive rate is very low. Conducting large sizes of n-grams yields approximately the same accuracy where it increases the computational cost. Accordingly, the 6-gram model seems to be the optimal initial n-gram size for the SSEs sequence matching procedure. As mentioned in the methods section, this procedure conducts a decreasing size of n-grams to match SSEs sequences.

**Table 4:** Accuracy index adopted from Receiver Operating Characteristic (ROC) curve

	3-gram	4-gram	5-gram	6-gram
				
TPR*	0.437	0.523	0.852	0.982
FPR*	0.129	0.103	0.053	0.024

* True Positive Rate (TPR) and False Positive Rate (FPR)

### Optimization of the alignment at the residue level

The procedure for the alignment at the residue level requires the optimization to choose an optimum gap penalty. This optimization is based on two contradictory criteria including the length of alignment and RMSD. Therefore, TM_score _was used as a balance between these measures to evaluate the quality of the alignment in this experiment. The test uses a randomly selected set of 1000 protein structures from the PDB with less than 40% sequence identity. In order to choose the optimal gap penalty, all-against-all structural alignment was performed at the following gap penalty values {-1.0, -2.0, -3.0, -4.0, -5.0, -6.0}, and the TM_score _was calculated for each alignment.

**Figure 5:**
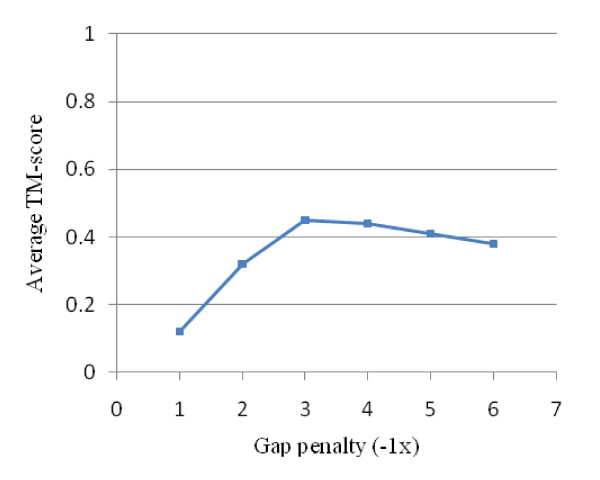
**Average TM_score _obtained at different gap penalties**.

Figure 5 shows the average TM_score _obtained at different gap penalty values. In this figure, a higher TM_score _value indicates a higher degree of precision and/or length in the alignment. As shown in the figure, increasing the negative gap penalty yields a higher TM_score _on varying gap penalties ranging from -1.0 to -3.0. Based on the figure, the optimal gap penalty value is -3, where for the higher values, the TM_score _decreases slightly. Indeed, the high gap penalty values prevent the alignment from extending along the protein structure, and yet the low value yields numerous gaps in the alignment with a low biological significance. Accordingly, the value of -3.0 seems to be the optimal gap penalty value in our experiment.

Additionally, we used the above dataset to test the optimality of the alignment procedure. The experiment was run by choosing different initial sets of fragments to make and apply a rotation matrix based on the alignment procedure. There are only 58 (≈6%) items with an alternative choice for initial fragment with a difference less than 0.01. Accordingly, the procedure is deemed sufficient based on the fast convergence of the rotation matrix after 3-4 repetitions.

### Benchmark study

The benchmark study was performed using a set of 200 non-homologous protein chains collected from the PDB by Zhang and Skolnick [7] with a pair wise sequence identity of less than 30%. The structures in the dataset are subject to comparison in an all-against-all approach by TS-AMIR and the results are compared with those of the CE, TM-align and 3D-BLAST programs. The results of CE and TM-align were taken from the literature [7]. A summary of the alignment results is represented in Table 5 including the averages over all 200 × 199 protein pairs.

**Table 5:** Alignment results summary for 200 non-homologous proteins averaged over all structure pairs

	Length of alignment	Coverage	RMSD (Å)	TM_score_
CE	64.3	34.7%	6.52	0.169
TM-Align	87.4	42.0%	4.99	0.253
3D-BLAST	65.7	36.2%	6.69	0.172
TS-AMIR	91.4	46.6%	6.17	0.237
				

- The results of CE and TM-Align were taken from [7].

- Coverage denotes fraction of residues aligned within the target protein.

- Length of alignment denotes number of aligned residues

The table shows the alignment accuracy by RMSD, the length of alignment, and coverage, which is the fraction of the aligned residues in the target protein. The results produced by CE are included in this table as a basic reference method to evaluate other alignment tools. As seen from the table, TS-AMIR has the largest length of alignment (91.4) and coverage (46.6%) where its accuracy in terms of RMSD (6.17) is ranked second, less than CE and 3D-BLAST. Moreover, TM-align is the best in terms of RMSD (4.99) where it obtains the second rank in the length of alignment (87.4) and coverage (42.0%). Furthermore, comparing the average TM_score _in Table 5 TM-align has the best rank (0.253) followed by TS-AMIR (0.237) with a low difference and 3D-BLAST (0.172).

Most of the structures collected in the dataset belong to different folds with low TM_score _values. In order to significantly compare the ability of the methods to match the most similar structure to a given target protein, the averages over only the highest TM_score _match for each target protein are computed in Table 6. In this table, TS-AMIR has the best rank in terms of coverage (74.7%) and the second rank in terms of TM_score _(0.502), while positioned lower than TM-align (0.510) showing a slight difference with the first rank.

**Table 6:** Alignment results summary for the same dataset in table 5 averaged over the most similar pairs

	Length of alignment	Coverage	RMSD (Å)	TM_score_
CE	128.8	61.4%	3.95	0.441
TM-Align	166.2	73.1%	4.45	0.510
3D-BLAST	131.4	63.1%	4.32	0.454
TS-AMIR	168.9	74.7%	4.48	0.502
				

- The results of CE and TM-Align were taken from [7].

### Evaluation of retrieval effectiveness

**Figure 6:**
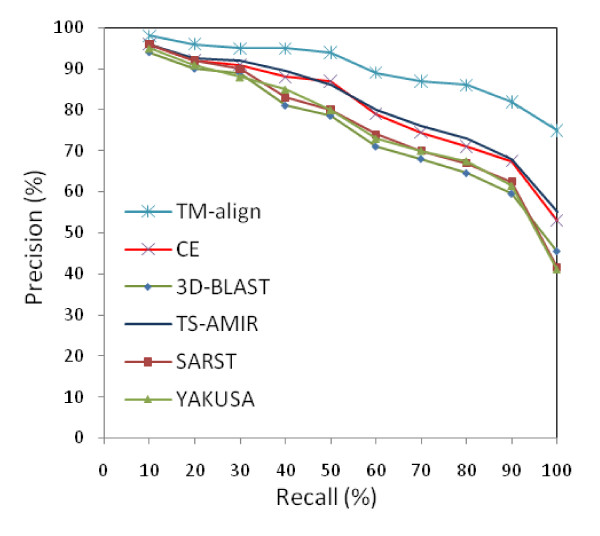
**Average precision-recall for searching 108 query proteins**.

The retrieval effectiveness of the schemes was investigated using Aung and Tan collected dataset [33] of 34,055 proteins from ASTRAL SCOP 1.59 along with the subset of 108 query proteins belonging to four major SCOP categories, All-Alpha, All-Beta, Alpha/Beta and Alpha+Beta. The efficiency of the methods was evaluated using the *precision *and *recall *parameters as two commonly used measures of information retrieval assessments. The parameters are determined as follows:

(7) Precision=m/n

(8) Recall=m/N

where *m *is the number of correct retrievals from the same SCOP family, *n *is the total number of retrieved proteins, and *N *denotes the total number of relevant proteins within the same SCOP family. The experiment uses the above dataset to search 108 query proteins, and the precision and recall values are evaluated for each method. The average precision-recall values calculated for the six different schemes are shown in Figure 6. The results of the methods except for TM-align and TS-AMIR were taken from the literature [22]. According to the figure, TM-align is in the first rank in terms of accuracy. In the second rank, TS-AMIR and CE are competitively accurate, although TS-AMIR obtains slightly better accuracy in the higher percentages. Moreover, three linear encoding schemes obtain generally lower accuracy than TS-AMIR.

The performance of the schemes in terms of running speed was also measured and compared in Table 7 where all the experiments were done on a 3.2 GHz CPU. As shown in Table 7 SARST obtains the best running time of the search within the database of 34,055 proteins. 3D-BLAST and TS-AMIR are in the second rank with approximately the same running speed, which is more than 7200, 800 and 3 times faster than CE, TM-align and YAKUSA, respectively.

**Table 7:** Average running time of the methods to search in a database of 34,055 proteins (in seconds)

Method	Average time per query	Average time per comparison
CE	82789.20	2.43
TM-align	9273.41	0.272
YAKUSA	35.60	0.00105
TS-AMIR	11.47	0.000337
3D-BLAST	9.07	0.000266
SARST	0.34	0.00000998

- Except for TM-align and TS-AMIR, the Results were taken from literature [22].

- The experiments were done on a 3.2 GHz CPU.

### Retrieval effectiveness on structural categories

The performance of the methods was evaluated in the determination of structural categories of each query structure from the above 108 proteins. To this end, the false positive rate was computed as a measure of the probability of irrelevant retrieval, where the lower rate indicates a higher degree of efficiency in the retrieval assessment.

**Figure 7:**
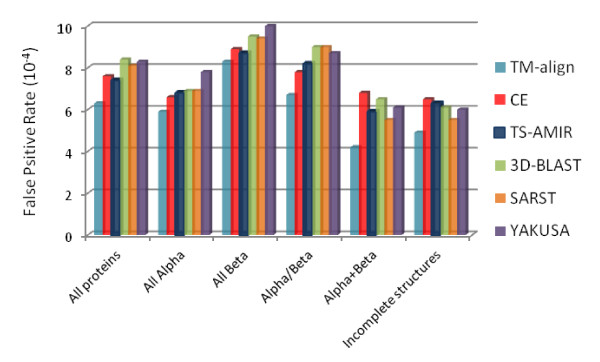
**Retrieval effectiveness on different structural categories**.

The average false positive rates were computed for four categories of the SCOP database after the retrieval of 80 proteins for each query structure. Figure 7 shows the results for the six schemes where the results except for TM-align and TS-AMIR were taken from the literature [22]. Based on the results in this figure, TS-AMIR yields false positives approximately similar to CE, but lower than those of SARST, YAKUSA and 3D-BLAST. TM-align obtains the best performance in this assessment.

Furthermore, a similar experiment was carried out using proteins with missing residues, known as incomplete structures [33], which constitute about one-fifth of the query proteins. The results in the last part of Figure 7 illustrate that TM-align is the best among the methods with considerably low false positives. Moreover, CE and TS-AMIR generate slightly more false positives for the incomplete structures than SARST, YAKUSA and 3D-BLAST. In general, the low efficiency of linear encoding methods is due to the weakness of these methods in converting structural details of incomplete backbone coordinates [19]. However, the ability of TS-AMIR to improve this efficiency is limited due to the incompatibility of the employed strategy in the alignment procedure at the residue level, as the missing residues are not taken into consideration.

### Retrieval effectiveness on low sequence identity

The efficiency of the above six methods were examined for homology searching in the low sequence identity. The experiment uses the dataset of 24,337 proteins from ASTRAL SCOP 1.69 collected by Lo *et al*. [22]. A subset of 83 query proteins belonging to the four main SCOP categories with certain features such as having the sequence identity of less than 10%, without missing residues and having at least two family members in the dataset, were selected and subtracted from the dataset (additional details about the dataset are available at Lo *et al*. [22]).

**Figure 8:**
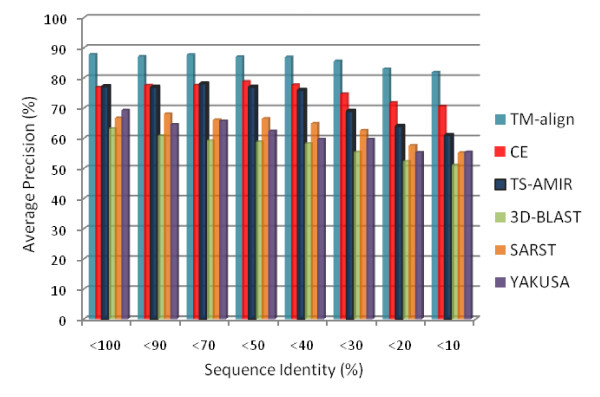
**Retrieval effectiveness on low sequence identity**.

The above dataset was utilized to perform an information retrieval experiment on a subset of query proteins. As shown in Figure 8, the methods display decreasing precision with decreasing sequence identity. TM-align obtains the highest precision in this test. Moreover, CE and TS-AMIR are competitively accurate for the sequence identities higher than 40%. However, the accuracy of TS-AMIR is lower than that of CE when sequence identities fell below 30%. Obviously, TM-align and CE as geometric methods are more precise than sequence-based methods. However, TS-AMIR improves this precision by focusing on the geometry of the structures in the alignment procedure at the residue level.

## Discussion

### In general

The main objective in this study was to develop a rapid protein structure alignment tool by combining two fruitful strategies: geometry-based approach and linear encoding approach. Indeed, TS-AMIR gains the advantages of two different strategies. In the first stage, it uses a linear encoded form of protein secondary structure to reach an initial correspondence map. Linear encoding schemes offer the advantage of fast running speeds. In the second stage, the method utilizes an iterative algorithm to create a rotation matrix using the geometry of aligned residues. Geometrical schemes obtain high performance in terms of accuracy. Accordingly, the method combines the above two schemes to achieve high running speeds as well as a high degree of precision.

TS-AMIR has been developed with some key features demonstrating its preference and competence. First of all, the method uses a simplified representation of the secondary structure elements in a topology string in order to match the backbone of two structures. This has a complexity reduction advantage to a one-dimensional problem where it requires a string matching procedure based on language modelling techniques. Moreover, the method adopts the n-gram modelling technique from computational linguistics, making it superior to the other formal language models to capture similarities in the topology strings. As another feature, this representation makes the method free from any parameter setting by the user. Classical structure alignment techniques such as dynamic programming often require a set of optional parameters to reach the best possible match. Moreover, at the residue level, the method applies the Kabsch method to generate the rotation matrix as an efficient well-known solution for the optimal alignment of two structures in an iterative heuristic procedure. The alignment is converged after 3-4 iterations. Consequently, the method obtains a comparable performance with other state of the art programs.

Secondary structure encoding in a 1D topology string provides several advantages in structural analysis and alignment of proteins. Firstly, it facilitates the representation of protein backbone structure in a summarized 1D-sequence making an economic pre-calculated database of the structural information. Secondly, the search algorithm to determine the initial overlap between two structures is sped up by choosing an efficient sequence alignment technique to match these strings. Thirdly, classifying the proteins into structural categories within large databases can be done efficiently. Finally, the scheme can be used to simplify computationally complex structure analysis tools by rapidly filtering out irrelevant structures.

### On running speed

As mentioned above, TS-AMIR adopts two relatively simple strategies from both linear encoding and geometry-based schemes to be used in secondary structure matching and residue level alignment respectively. Therefore, TS-AMIR obtains a competitive running speed in comparison with the other rapid structure alignment techniques.

Comparing the running time of the methods to search for each query protein shows that TS-AMIR runs 3 times faster than YAKUSA, approximately as fast as 3D-BLAST, and more than 30 times slower than SARST method. The lower speed of TS-AMIR against SARST seems to be due to utilizing the iterative procedure for the alignment at the residue level by TS-AMIR in order to make and apply the rotation matrix. However, TS-AMIR is more precise compared to SARST in the information retrieval assessments. Considering the high speed of linear encoding schemes, they are mostly developed as a search tools to run queries in the large structure databases. Therefore, they ignore giving highly accurate results in favour of reaching a high running speed. TS-AMIR improves the low accuracy of the linear encoding approaches by using a geometry-based technique for the alignment at the residue level.

### On alignment accuracy

Although TS-AMIR did not yield a highly accurate alignment like TM-align, it has shown a competitive accuracy with CE which is known to be a highly accurate method. Moreover, TS-AMIR outperforms the other linear encoding methods such as YAKUSA, 3D-BLAST and SARST in terms of accuracy.

Looking at the results of the benchmark test, it is clearly visible that TS-AMIR obtains high alignment quality in terms of TM_score _after TM-align. The high efficiency of the method is yielded by the high length of alignment with a relatively low RMSD. This is due to the strategy of the method for the alignment of residues in an iterative step-by-step algorithm. The method first looks for small fragments within matched SSEs to make an initial alignment, and then extends the alignment to the rest of the residues by considering a penalty for each gap. The allowed gaps between the aligned residues provide an opportunity to superpose large and significant coverage between the two structures. Moreover, the algorithm clears the alignment list for each repetition, and looks again for the alignment. This helps the algorithm to determine a more accurate alignment after applying the rotation matrix at the end of each repetition. Due to the convergence of the rotation matrix after 3-4 attempts, these iterations do not critically increase the running time of the algorithm.

Structure alignment programs using a hierarchical approach ranging from secondary structure matching to the atomic superposition level [6,28] are usually along with translating the SSEs matching results to the alignment of residues. The SSM method [6], as a well-known hierarchy oriented structure alignment tool, is comparatively weak in making a relationship between the topology of the SSEs, which is considered as an important feature in the determination of common scaffolds in distantly related structures. TS-AMIR uses a reasonable strategy for encoding the geometry of SSEs in a topology string by assuming inter-SSE vectors reflecting the relative position of two consecutive SSEs to the string. Moreover, the algorithm for matching two topology strings of compared proteins chooses a decreasing size of the n-gram model starting from 6 as an empirically chosen parameter. Therefore, topological relationships between the SSEs are effectively considered in the SSEs matching procedure. However, methods using the secondary structure to find primarily an initial overlap between two structures often perform poorly when the secondary structure of an input query is not strongly provided. TS-AMIR also needs well-defined secondary structure details of the query proteins in order to yield highly accurate alignment results.

### On information retrieval

The results of assessments for information retrieval make evident that geometry-based methods are preferred to linear encoding methods. Taking advantage of both schemes, TS-AMIR shows improved accuracy in the assessments similar to that of CE, but better than those of other linear encoding programs.

Nevertheless, based on the experimental results, the method displays occasional weakness in the retrieval of relevant structures. After subjecting the method to several irrelevant retrievals, it was illustrated that the weakness is due to the repetition of common SSE subsequences shared by different proteins within the whole structure. In fact, TS-AMIR uses n-gram modelling to match the subsequences of SSEs topology strings. Therefore, the method occasionally falls in ambiguity for matching the n-grams, which is in accordance with its multiple occurrences along the topology string, thus leading to the applied heuristic's failure in making decision. However, by setting a relevant n-gram size in the SSEs matching procedure, these irrelevant retrievals are minimized.

Additionally, TS-AMIR achieves a fair performance on retrieval of structural categories among the methods where it produces low false positives for different SCOP categories. This is conceptual due to the adoption of an efficient strategy for matching SSEs based on the topology strings. It was expected that the method would produce more false positives due to repetition of alpha helices along with the protein chain as the most abundant regular element of secondary structure. Therefore, the probability of irrelevant matching between SSEs is increased. However, the method overcomes this expectation and yields fair results by choosing the appropriate decreasing sizes of n-gram as mentioned in the methods section.

## Conclusion and future work

We have developed a rapid protein structure alignment tool called TS-AMIR, a Topology String Alignment Method for Intensive Rapid Protein Structure Comparison, which is a combination of a linear encoding scheme in the first stage and a geometry based technique in the second stage. In terms of speed, the experimental results demonstrate the high performance of the method as it performs as well as linear encoding schemes. In addition, the method obtains results as highly accurate as the geometry based approaches. This high efficiency results from the simple and efficient techniques which are employed by the method.

Further studies will focus on the application of the approach for non-sequential structural alignment of proteins, which needs to neglect connectivity of the polypeptide chains. Considering the flexibility of the strategy applied by TS-AMIR for the superposition of the secondary structure elements by topology strings, the approach seems to be relevant for use in the detection of non-sequential structural analogies in the proteins. Moreover, recent studies to fuse theoretical concepts from computational linguistics in structural biology motivate the researchers to conduct further studies in this area.

## Competing interests

The authors declare that they have no competing interests.

## Authors' contributions

JR carried out the design and development of the algorithm, performed the experiments and their statistical analysis, and drafted the manuscript. SD carried out the design and supervision of the study. SP participated in data collection and analysis, and helped to draft the manuscript. All authors read and approved the final manuscript.

## References

[B1] Shibuya T Jansson J Sadakane K Linear-time protein 3-D structure searching with insertions and deletions Algorithms for Molecular Biology 2010 5 7 10.1186/1748-7188-5-7 20047663 PMC2830924

[B2] Holm L Sander C Protein structure comparison by alignment of distance matrices Journal of Molecular Biology 1993 233 123 138 10.1006/jmbi.1993.1489 8377180

[B3] Gibrat JF Madej T Spouge JL Bryant SH The VAST protein structure comparison method Biophysics Jounnal 1997 72 MP298

[B4] Shindyalov I Bourne P Protein structure alignment by incremental combinatorial extension (CE) of the optimal path Protein Engineering 1998 11 739 47 10.1093/protein/11.9.739 9796821

[B5] Ortiz AR Strauss CE Olmea O MAMMOTH (matching molecular models obtained from theory): an automated method for model comparison Protein Science 2002 11 2606 2621 12381844 10.1110/ps.0215902 PMC2373724

[B6] Krissinel E Henrick K Secondary-structure matching (SSM), a new tool for fast protein structure alignment in three dimensions Acta Crystallographica Section D: Biological Crystallography 2004 60 2256 2268 10.1107/S0907444904026460 15572779

[B7] Zhang Y Skolnick J TM-align: a protein structure alignment algorithm based on the TM-score Nucleic Acid Research 2005 33 2302 2309 10.1093/nar/gki524 PMC1084323 15849316

[B8] Kolbeck B May P Schmidt-Goenner T Steinke T Knapp EW Connectivity independent protein-structure alignment: a hierarchical approach BMC Bioinformatics 2006 7 doi:10.1186/1471-2105-7-510 10.1186/1471-2105-7-510 PMC1683948 17118190

[B9] Kawabata T MATRAS: a program for protein 3D structure comparison Nucleic Acids Research 2003 31 3367 3369 10.1093/nar/gkg581 12824329 PMC168987

[B10] Sierk ML Kleywegt GJ DEJAVU All Over Again: Finding and analyzing Protein Structure Similarities Structure 2004 12 2103 2111 15576025 10.1016/j.str.2004.09.016

[B11] Mosca R Schneider T RAPIDO: a web server for the alignment of protein structures in the presence of conformational changes Nucleic Acids Research 2008 36 Web Server W42 W46 10.1093/nar/gkn197 PMC2447786 18460546

[B12] Salem S Zaki MJ Bystroff C FlexSnap: Flexible Non-sequential Protein Structure Alignment Algorithms for Molecular Biology 2010 5 12 10.1186/1748-7188-5-12 20047669 PMC2846951

[B13] Mayr G Domingues F Lackner P Comparative analysis of protein structure alignments BMC Structural Biology 2007 7 50 doi: 10.1186/1472-6807-7-50 10.1186/1472-6807-7-50 17672887 PMC1959231

[B14] Kolodny R Koehl P Levitt M Comprehensive evaluation of protein structure alignment methods: scoring by geometric measures Journal of Molecular biology 2005 346 1173 1188 10.1016/j.jmb.2004.12.032 15701525 PMC2692023

[B15] Novotny M Madsen D Kleywegt GJ Evaluation of protein fold comparison servers Proteins: Structure, Function, and bioinformatics 2004 54 260 270 10.1002/prot.10553 14696188

[B16] Pearson WR Rapid and sensitive sequence comparison with FASTP and FASTA Methods in enzymology 1990 183 63 98 2156132 10.1016/0076-6879(90)83007-v

[B17] Altschul SF Madden TL Schaffer AA Zhang J Zhang Z Miller W Lipman DJ Gapped BLAST and PSI-BLAST: a new generation of protein database search programs Nucleic Acids Research 1997 25 3389 3402 10.1093/nar/25.17.3389 9254694 PMC146917

[B18] Martin ACR The ups and downs of protein topology; rapid comparison of protein structure Protein Engineering 2000 13 829 837 10.1093/protein/13.12.829 11239082

[B19] Guyon F Camproux AC Hochez J Tuffery P SA-Search: a web tool for protein structure mining based on a structural alphabet Nucleic Acids Research 2004 32 W545 W548 10.1093/nar/gkh467 15215446 PMC441605

[B20] Carpentier M Brouillet S Pothier J YAKUSA: a fast structural database scanning method Proteins 2005 61 137 151 10.1002/prot.20517 16049912

[B21] Tung CH Huang JW Yang JM Kappa-alpha plot derived structural alphabet and BLOSUM-like substitution matrix for rapid search of protein structure database Genome Biology 2007 8 R31 10.1186/gb-2007-8-3-r31 17335583 PMC1868941

[B22] Lo WC Huang PJ Chang CH Lyu PC Protein structural similarity search by Ramachandran codes BMC Bioinformatics 2007 8 307 10.1186/1471-2105-8-307 17716377 PMC2194796

[B23] Bauer RA Rother K Moor P Reinert K Steike T Bujnicki JM Preissner R Fast structural alignment of biomolecules using hash table, n-gram and string descriptors Algorithms 2009 2 692 709 10.3390/a2020692

[B24] Budowski-Tal I Nov Y Kolodny R FragBag, an accurate representation of protein structure, retrieves structural neighbors from the entire PDB quickly and accurately PNAS 2010 107 3481 3486 10.1073/pnas.0914097107 20133727 PMC2840415

[B25] Razmara J Deris SB A novel text modelling approach for structural comparison and alignment of biomolecules WSEAS Transactions on Computers 2010 9 675 685

[B26] Kabsch W A solution for the best rotation to relate two sets of vectors Acta Crystallographica Section A: Crystal Physics, Diffraction, Theoretical and General Crystallography 1976 32 922 923 10.1107/S0567739476001873

[B27] Kabsch W A discussion of the solution for the best rotation to relate two sets of vectors Acta Crystallographica Section A: Crystal Physics, Diffraction, Theoretical and General Crystallography 1978 34 827 828 10.1107/S0567739478001680

[B28] Singh AP Brutlag DL Hierarchical protein structure superposition using both secondary structure and atomic representations Proceedings of International Conference on Intelligent Systems in Molecular Biology 1997 5 284 293 9322051

[B29] Bogan-Marta A Hategan A Pitas I Language engineering and information theoretic methods in protein sequence similarity studies Studies in Computational Intelligence 2008 85 151 183 10.1007/978-3-540-75767-2_8

[B30] Zhang Y Skolnick J Scoring function for automated assessment of protein structure template quality Proteins 2004 57 702 710 10.1002/prot.20264 15476259

[B31] Liao L Noble W Combining pairwise sequence similarity and support vector machines for remote protein homology detection Journal of Computational Biology 2003 10 857 868 10.1089/106652703322756113 14980014

[B32] Chionh CH Huang Z Tan KL Yao Z Augmenting SSEs with structural properties for rapid protein structure comparison Proceedings of 3rd IEEE Symposium on BIBE 2003

[B33] Aung Z Tan KL Rapid 3D protein structure database searching using information retrieval techniques Bioinformatics 2004 20 1045 1052 10.1093/bioinformatics/bth036 14962928

